# Trends and Racial and Geographic Differences in Infant Mortality in the United States Due to Necrotizing Enterocolitis, 1999 to 2020

**DOI:** 10.1001/jamanetworkopen.2023.1511

**Published:** 2023-03-03

**Authors:** Mattie F. Wolf, Allison T. Rose, Ruchika Goel, Jennifer Canvasser, Barbara J. Stoll, Ravi M. Patel

**Affiliations:** 1 Division of Neonatology, Department of Pediatrics, Emory University School of Medicine and Children’s Healthcare of Atlanta, Atlanta, Georgia; 2 Division of Hematology/Oncology, Department of Internal Medicine, Simmons Cancer Institute, Springfield, Illinois; 3 NEC Society, Davis, California

## Abstract

This cohort study analyzes yearly trends in necrotizing enterocolitis–related infant mortality rates (NEC-IMR) from 1999 to 2020, overall and by Black and White race, and described Black-to-White NEC-IMR ratios and NEC-IMR for US states.

## Introduction

Necrotizing enterocolitis (NEC) is the most common cause of death between 2 weeks and 2 months of age in extremely preterm infants, and the only cause that increased from 2000 to 2011.^1^ By contrast, another study reported declines in NEC from 2006 to 2017.^2^ Analysis of population-based trends in NEC-related infant mortality rates (NEC-IMR) spanning these periods is lacking. Additionally, Black race^3,4^ and Southeastern US geographic location^5^ have previously been associated with higher NEC-IMR, but recent estimates by race and region are unknown. We analyzed yearly trends in NEC-IMR from 1999 to 2020, overall and by Black and White race, and described Black-to-White NEC-IMR ratios and NEC-IMR for US states.

## Methods

This cohort study analyzed data from the Centers for Disease Control and Prevention and National Center for Health Statistics’ 2020 Final Multiple Cause of Death Data and followed the STROBE reporting guideline. This project was determined to not require review or informed consent by the Emory University Institutional Review Board as it was not considered research with human participants, in accordance with 45 CFR §46. We evaluated all infant deaths up to 1 year of age with the underlying cause of NEC of newborn (*International Statistical Classification of Diseases and Related Health Problems, Tenth Revision [ICD-10]* code P77). NEC-IMR was calculated per 100 000 live births. Race was ascertained based on death certificate reporting and American Indian or Alaskan Native and Asian/Pacific Islander were not examined due to small numbers (less than 10 deaths), per data use guidance.

Joinpoint regression version 4.7.0.0 (National Cancer Institute) with a max of 3 joinpoints (to allow approximately 7 years per joinpoint) was used to examine NEC-IMR by year, including stratification by Black vs White race, to identify points where linear trends changed significantly in either magnitude or direction. We used tests of parallelism to determine if the mean segmented lines by race were parallel or not and the Poisson distribution to calculate 95% CIs. We used linear regression (GraphPad Prism 9 [Dotmatics]) to compare Black-to-White NEC-IMR ratios by year and state NEC-IMR across all years, excluding states with less than 10 deaths. *P* < .05 determined statistical significance.

## Results

**Figure 1.**
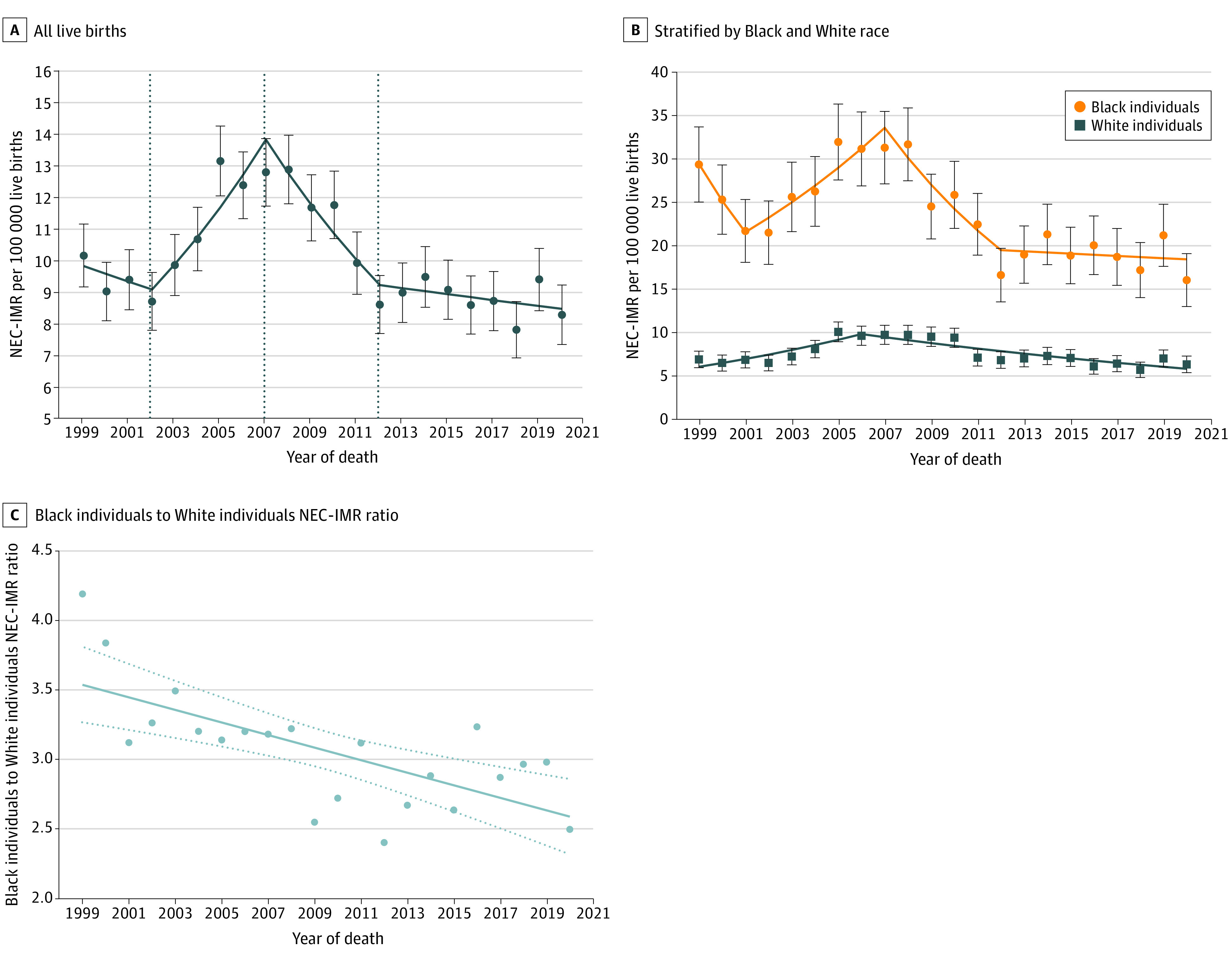
Trends in Necrotizing Enterocolitis(NEC)–Related Infant Mortality Rate (NEC-IMR) From 1999 Through 2020 Dots in panels A and B indicate crude NEC-IMR estimates and whisker bars indicate 95% CIs. In panel C, the dots indicate the Black-to-White NEC-IMR ratio, the solid line is a fitted linear regression line, and dotted lines indicate 95% CIs.

**Figure 2.**
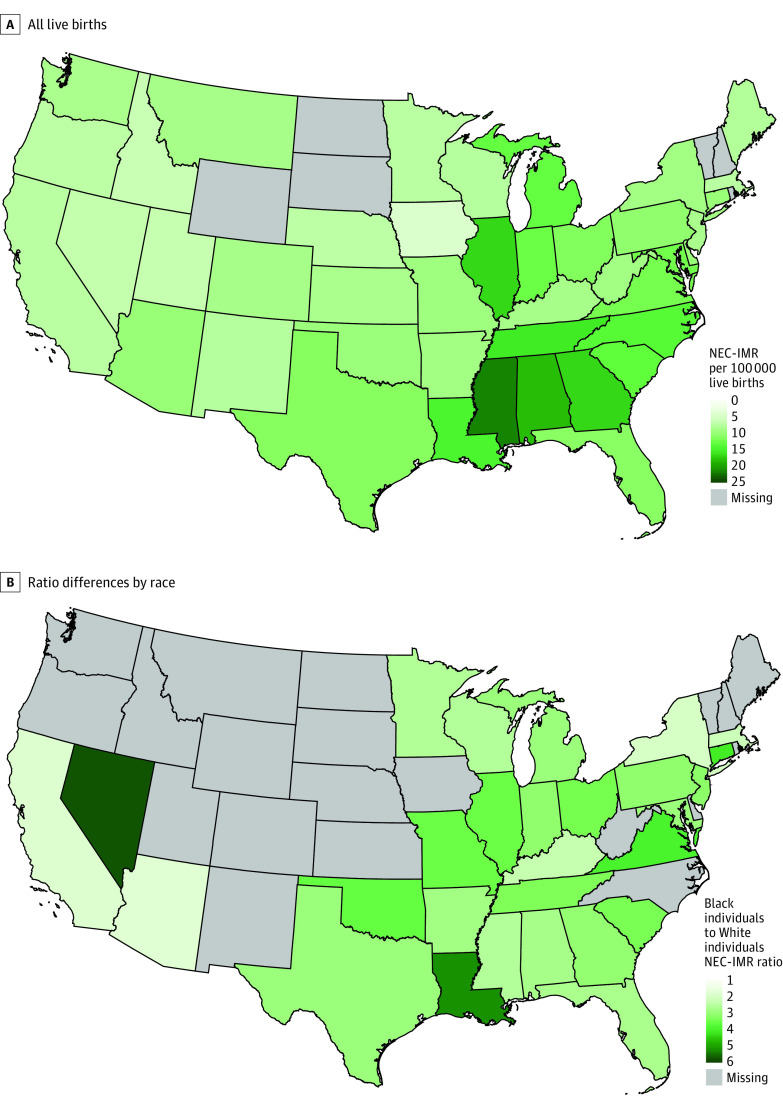
Necrotizing Enterocolitis–Related Infant Mortality Rate (NEC-IMR) by US State A, The 1999-2020 NEC-IMR by each state with the colorimetric scale indicating NEC-IMR per 100 000 live births. B, Racial differences in NEC-IMR, with the colorimetric scale indicating the Black-to-White ratio of NEC-IMR. States with fewer than 10 deaths per race are not reported and shaded in gray, except Alaska. Hawaii’s NEC-IMR was 7.0.

Among 88 125 233 live births from 1999 to 2020, 8951 infants died from NEC (overall NEC-IMR across all years 10.2 [95% CI, 10.0-10.4] per 100 000 live births [For 2020, Black: 16.1 (95% CI, 13.1-19.2) per 100 000; White: 6.4 (95% CI, 5.5-7.4) per 100 000]). The NEC-IMR had an inflection in trend in 2007, with a decline of 7.7% (95% CI, 2.1%-13.0%) per year through 2012, with no significant decline thereafter (Figure 1A). The NEC-IMR peaked in 2005 at 13.2 (95% CI, 12.1-14.3) per 100 000 live births and was lowest in 2020 at 8.3 (95% CI, 7.4-9.3) per 100 000 live births. Segmented lines were not parallel for Black infants compared with White infants (*P* = .04 from test of parallelism) (Figure 1B). The Black-to-White NEC-IMR ratio declined over the study period from 4.2 in 1999 to 2.5 in 2020 (*P* = .007, Figure 1C). The 1997 to 2020 NEC-IMR ranged from 22.0 (95% CI, 18.9-25.1) per 100 000 live births in Mississippi to 4.4 (95% CI, 3.1-6.0) per 100 000 live births in Iowa (Figure 2A). The Black-to-White NEC-IMR ratio across all years by state ranged from 1.8 to 5.8 (Figure 2B). There was no association between a state’s overall NEC-IMR and the Black-to-White NEC-IMR ratio.

## Discussion

This cohort study is compatible with findings from prior reports showing both increases^1^ and subsequent decreases^2^ in NEC-related deaths. Additionally, striking differences in Black-to-White NEC-IMR decreased over time, and a state’s Black-to-White ratio was unrelated to its NEC-IMR. Racial differences mirror overall disparity in IMR. Further studies are warranted to examine factors mediating these changes, including the role of increasing donor human milk use.^6^ Limitations include potential misclassification of the cause of death, exclusion of some states due to small numbers, and lack of disease classification beyond *ICD-10* diagnosis. These data may support clinicians, patients, families, and policy makers in understanding national trends in NEC to inform research, care practices, and policies.

## Notes

Supplement.
Data Sharing Statement
