# Supplementary material for: Trends and Racial and Geographic Differences in Infant Mortality in the United States Due to Necrotizing Enterocolitis, 1999 to 2020
Source: JAMA Netw Open. 2023 Mar 3;6(3):e231511. doi: 10.1001/jamanetworkopen.2023.1511 (PMC9984971; doi:10.1001/jamanetworkopen.2023.1511)
Supplement: Supplement. — Data Sharing Statement [file jamanetwopen-e231511-s001.pdf]

Data Sharing Statement

Wolf. Trends and Racial and Geographic Differences in Infant Mortality in the United States Due to Necrotizing Enterocolitis, 1999 to 2020. *JAMA Netw Open*. Published March 03, 2023. doi:10.1001/jamanetworkopen.2023.1511

Data
Data available: Yes
Data types: Other (please specify)
Additional Information: Source data are publicly available.
How to access data: https://wonder.cdc.gov/
When available: With publication

Supporting Documents
Document types: None

Additional Information
Who can access the data: Anyone requesting the data
Types of analyses: For any purpose
Mechanisms of data availability: Without investigator support
Any additional restrictions: None
